# Parents’ roles in young learners’ motivation and task engagement in Indonesian primary schools: Questionnaire development and validation

**DOI:** 10.12688/f1000research.170391.1

**Published:** 2025-10-17

**Authors:** Mai Sri Lena, Marianne Nikolov

**Affiliations:** 1 Doctoral school of education, University of Szeged, Szeged, H-6720, Hungary; 2 Department of Elementary School Teacher Education, Universitas Negeri Padang, Padang, 25171, Indonesia; 3 Institute of English Studies, University of Pécs, Pécs, H-7622, Hungary

**Keywords:** Parents’ role, young learners, motivation, task engagement, primary school, validation

## Abstract

**Background:**

Parental involvement in a child’s second language learning is important because it affects the process and outcomes. No research has been conducted in the Indonesian context on what roles parents play in their children’s motivation and task engagement.

**Methods:**

This study aimed to develop and validate an instrument to measure how parents impact young learners’ motivation to learn English and to engage with tasks. Participants were 270 parents of fifth graders learning English at nine public and private schools in Padang. The instrument was developed by analysing the literature and existing tools and creating new items. After getting expert feedback and piloting the survey, we assessed its validity and reliability. Research questions examined factors affecting its effectiveness.

**Results:**

The questionnaire was analysed through EFA and CFA via jamovi. EFA identified five dimensions: (1) parental involvement, (2) expectations, (3) access to resources, (4) enrichment and (5) extracurricular activities. The CFA fit indices (CFI = .945, TLI = .934, SRMR = .045, RMSEA = .059) confirmed the model’s suitability. The questionnaire showed strong validity and reliability, with measures exceeding.70, making it effective for gathering data on parental roles in Indonesian children’s learning of English.

**Conclusions:**

This validation study offers an effective diagnostic tool for teachers, administrators, and policymakers to pinpoint the particular dimensions of parental involvement that affect children’s motivation and task engagement as they learn English. The findings highlight the critical role of recognising parents as active collaborators along the language learning journey. The study improves the theoretical understanding of the impact of parental behaviour in educational psychology and motivation studies. The findings are consistent with self-determination theory, providing a more nuanced perspective to explore how different forms of parental participation influence student motivation and task engagement in learning English.

## Introduction

The study aims to develop, pilot, and validate a questionnaire on the role of parents in motivating and engaging young learners with the tasks assigned by teachers in their English classes. Young learners’ (YLs) motivation to learn English is influenced by internal and external factors, including their interest in English, interactions with teachers, peers, and parents, classroom environment, and societal value on English. In Indonesia, where English is a foreign language, motivation is crucial for YLs to continue and enjoy learning despite limited exposure outside school. Indonesian children often start learning English early, making it vital to nurture motivation for long-term language development. Understanding motivation development is key to forming effective teaching methods and addressing challenges such as unequal access to instruction and varying levels of parental involvement. Research shows that motivation significantly affects learners’ engagement, persistence, and achievement in language learning ( Lamb, 2017), making it essential for improving English education in Indonesia.

According to the Ministry of Education and Culture Regulation Number 51 of 2018 on the admission of new students to primary schools in Indonesia, YLs are 7-12-year-old pupils in primary school. Given English’s status as a global language, introducing it to young learners at an early stage is advantageous for their exposure to the language. Therefore, English is a compulsory subject for students in Elementary schools starting from grade three, based on Ministerial Regulation Number 12 of 2024 concerning the curriculum in early childhood education, elementary education level, and secondary education level. Some schools provide an English subject to their students from grade one to six, and some others from grade three to six, depending on the schools’ decision. However, little research has been conducted on YLs’ needs, who and what motivates them to learn English at the primary school level. Therefore, there is a need to investigate this topic further.

The literature revealed that parental involvement in a child’s second language learning is important because it affects children’s motivation (e.g., Choi et al., 2024; Liang et al., 2024; Mihaljević Djigunović & Nikolov, 2019) and engagement (e.g., Fan & Williams, 2010; Liang et al., 2024). However, no empirical studies have explored how parents perceive their role in their children’s learning of English at the primary school level. Existing questionnaires focus on students ( Butler & Le, 2018; Liu et al., 2016; Oga-Baldwin et al., 2017; Wang & Bai, 2023) and teachers ( Kalayci & Ergül, 2020) and are designed for Western contexts, making them less suitable for use with Indonesian parents of young learners.

This research introduces a novel approach by bridging a contextual and methodological gap, presenting the first validated survey tailored to capturing Indonesian parents’ views on their role in enhancing their children’s motivation and task engagement in learning English. This research, by anchoring the instrument in the specific educational and cultural context, delivers unique data at a crucial moment—right before English is mandated as a core subject from an early level in all Indonesian primary schools. The results aim to academically augment motivation studies in less-explored settings and to practically guide improvements in English education by promoting greater family participation, especially the involvement of parents.

This study aimed to answer two research questions:

1. What are the underlying factors that structure the questionnaire measuring parents’ roles in their children’s English learning motivation and task engagement?
2. How valid and reliable is the questionnaire piloted for parents of fifth-grade learners of English?

Before designing the questionnaire, we reviewed the literature. In the first section, we explain how we decided which working definitions to use for various constructs related to how parents impact their children’s English language learning motivation and task engagement, how these were researched, and what the main findings were. Then, we outline the key variables we assumed to impact Indonesian young learners’ motivation and engagement, how we developed a survey for their parents, and how the data collection instrument worked with 270 participants in a pilot study.

## Literature review

### Parents’ roles in English language learning

Parental involvement is crucial in children’s English learning, influencing motivation and engagement ( Liang et al., 2024) and enhancing academic passion, study habits, and grades ( Shebani et al., 2025). Research indicates parental socioeconomic status (SES) combines education, income, and occupation ( Ma et al., 2023; Sirin, 2005) and positively correlates with L2 achievements ( Ma et al., 2023), academic results ( Balsa et al., 2018), and English learning ability ( Butler & Le, 2018).

University-educated mothers positively influenced their children’s English proficiency ( Jalili, 2017; Tan & Caleon, 2022). Low parental education correlated with higher primary education drop-out rates ( Ahsan et al., 2015). Family income affected children’s English learning engagement, with low-income children less engaged than those from middle-income families ( Poon, 2020).

**Figure 1.**
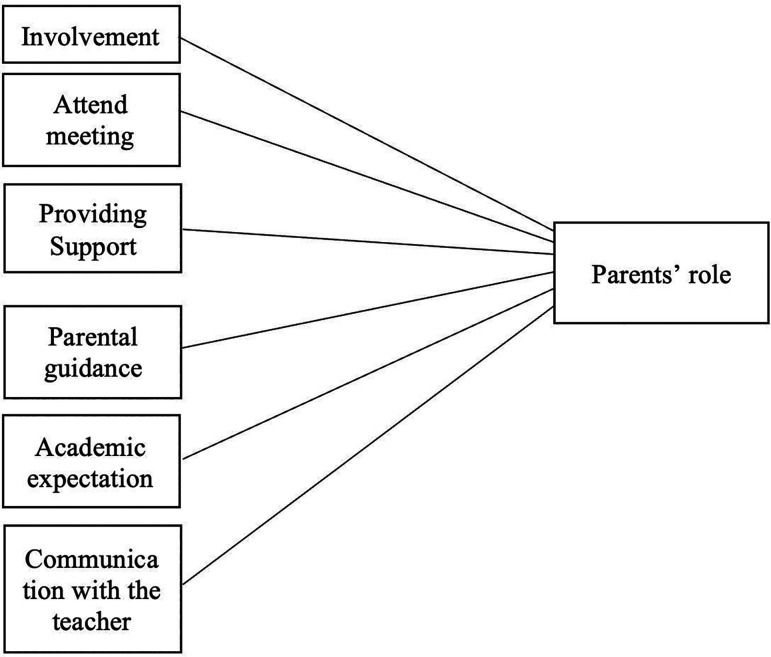
Parents’ role construct.

Various research papers have assessed the role of parental involvement in enhancing children’s academic achievements. The engagement of parents in their children’s English studies shows a significant link to the children’s proficiency in the language ( Butler & Le, 2018). Support provided by parents at home plays a crucial role in boosting students’ academic performance ( Tan & Caleon, 2022; Torrecilla & Hernández-Castilla, 2020). According to Kim et al. (2020), the psychological and behavioral participation of parents has a more profound effect on the motivation and accomplishments of children than the parents’ educational background. Communication between parents and teachers has been shown to augment students’ achievements ( Tan & Caleon, 2022) as well as their engagement and motivation to master English ( Fan & Williams, 2010). Involvement of parents in extracurricular activities and attendance at parent meetings has a notable influence on student performance ( Torrecilla & Hernández-Castilla, 2020). Additionally, parental guidance is a positive predictor of students’ intrinsic motivation to advance their English skills ( Fan & Williams, 2010), while parents’ academic expectations align positively with students’ English proficiency ( Butler & Le, 2018), achievements ( Tan & Caleon, 2022), engagement levels, and intrinsic motivation in English ( Fan & Williams, 2010). Figure 1 illustrates some parents’ roles in young learners’ English learning based on the literature.

### Motivation and task engagement in EFL contexts

Motivation is identified as a complex entity, consisting of three components: the aspiration to learn the language, perceptions regarding language learning, and the intensity of motivation demonstrated by continued effort in language acquisition ( Gardner, 2010). In English language learning, especially at the primary school level, motivation has been shown to influence learners’ persistence, participation, and language outcomes ( Dörnyei & Ushioda, 2011). Motivation plays a pivotal role in language learning; it determines the rate and success of acquiring a new language ( Gardner, 1985). This study conceptualizes motivation as the learners’ willingness, interest, and enthusiasm toward learning English. This study is based on self-determination in which motivation is divided into two types: intrinsic and extrinsic motivation ( Deci & Ryan, 1985; Ryan & Deci, 2020). **Intrinsic motivation** is defined as the interest or enjoyment in English tasks, while **extrinsic motivation** is encouraged by external rewards, pressures, or outcomes. These types of motivation are widely used in educational psychology and are appropriate for young learners’ development.

Task engagement refers to students’ involvement in completing tasks during the process of language learning ( Philp & Duchesne, 2016). It is understood as a construct with four distinct dimensions: behavioral, cognitive, emotional, and social engagement ( Philp & Duchesne, 2016). Behavioral engagement involves learners actively participating in tasks ( Philp & Duchesne, 2016), which can be assessed through factors like time dedicated to tasks and responding to questions ( Dörnyei & Kormos, 2000; Oga-Baldwin, 2019). Emotional engagement encompasses learners’ emotional connections and reactions to class activities ( Sang & Hiver, 2021). Cognitive engagement relates to alertness, continuous attention, active mental processes, mental effort, and self-regulation techniques ( Dao, 2021). Agentic engagement is defined by learners’ actions aimed at influencing classroom instruction ( Oga-Baldwin, 2019). Social engagement refers to the extent to which a student follows written and unwritten classroom rules of behavior, for example, coming to school and class on time, interacting appropriately with teachers and peers ( Finn & Zimmer, 2012).

Parents play a significant role in motivating young learners to learn English and in their engagement with tasks. Research indicates that parental involvement has a notable effect on children’s motivation ( Choi et al., 2024; Liang et al., 2024; Sumanti & Muljani, 2021; Tong et al., 2021) and engagement ( Liang et al., 2024). Interaction between parents and teachers is shown to enhance students’ motivation and engagement in English learning ( Finn & Zimmer, 2012).

### Parental questionnaires on young learners’ motivation and task engagement

Self-report questionnaires are the most frequently used tools to assess motivation and engagement in tasks. Some prior studies developed and adapted questionnaires to investigate motivation and engagement in tasks. For instance, Butler (2015b) and Butler and Le (2018) used parental survey that encompassed various aspects related to their children’s learning of the English language, which include: (a) the socioeconomic status (SES) of parents, such as household income and educational achievement levels of the parents; (b) indirect actions by parents aimed at supporting English learning, for instance, fostering a literacy-rich home environment and utilizing English at their workplace; (c) direct parental actions to facilitate English education, such as engaging in their children’s study process and arranging for private English tutoring post-school hours; (d) parental beliefs regarding English education, including the significance of English; and (e) specific beliefs parents have about their own child, like their expectation concerning the child’s success in learning English.

Torrecilla and Hernández-Castilla (2020) used a parental survey concerning household assets, their connection with the student and school, and their general contentment with the school.

Wang et al. (2023) used the Chinese version of parental involvement scale adapted from previous study containing 23 items across three aspects: personal, cognitive, and behavioral involvement. Personal involvement, with 9 items, measures perceived care about school life and emotions (e.g., easing emotions during learning difficulties). Cognitive involvement, with 8 items, assesses exposure to stimulating activities and materials (e.g., buying study materials). Behavioral involvement, with 6 items, evaluates parents’ participation in school activities (e.g., controlling online time). Both children and parents rated each item from “1 = never” to “5 = very often”, with higher scores indicating greater involvement.

However, no research has been conducted in the Indonesian context on the development and validation of instruments to measure parents’ involvement in their children’s motivation and engagement with tasks. Therefore, it is necessary to develop and validate a questionnaire on young learners’ motivation and task engagement from the perspectives of their parents. By doing so, it aims to enhance local educational practices and add to the existing body of knowledge in English language learning research at a broader level.

## Method

### Research design

The research study was quantitative in nature, meaning it involved the measurement and analysis of numerical data ( Creswell & Creswell, 2018). Its main focus was on the development and validation of a research tool or instrument. The study took place in primary schools in Indonesia. To gather data, researchers used questionnaires that were administered to participants. We used a convenience sampling technique in selecting the sample. The collected data were then analyzed using the statistical software program, jamovi app version 2.3.28.0 ( The jamovi project, 2022). This tool assists in performing complex data analyses and deriving meaningful conclusions from the collected data.

### Context of the study

The research was carried out in Padang, Indonesia’s elementary schools, where English is taught as a foreign language and is a required subject for students at elementary school since grade 3. Students in both private and public elementary schools learn English for 70 minutes a week with English teachers who have an English educational background.

### Participants

The study involved 270 parents of fifth-grade students, and their characteristics can be found in Table 1. The majority of respondents in this study were female (88.5%), with 48.9% aged between 38 and 44. Most parents (46.3%) reported their English proficiency as intermediate level, and they occasionally used English. They used it primarily for entertainment purposes (46.96%). More than half of the participants had a high school educational background (60.4%) and were housewives (64.4%). Then, 43% of respondents had a monthly income between IDR 1.000.000 and 5.000.000, while 38.1% chose not to disclose their incomes.

**Table:** 

English proficiency	Beginner	64	23.7

**Table 1.** Description of participants in this study.

Personal information	Category	Frequency	Percentage (%)
Gender	Male	31	11.5
	Female	239	88.5
Age	31-37	78	37.1
	38-44	132	48.9
	45-51	48	17.8
	52-58	8	3
	59-65	4	1.5
English proficiency	Low	64	23.7
	Lower-intermediate	73	37
	Intermediate	125	46.3
	Advanced	8	3
Using English	Regularly	58	21.5
	Sometimes	107	39.6
	Rarely	101	37.4
	Never	4	1.5
What for	Use it in my job	13	19.69
	Use it for entertainment	31	46.96
	Use it for my interest	12	18.18
	Use it for travel	10	15.15
Education background	High school	163	60.4
	Diploma degree	25	9.3
	Bachelor degree	60	22.2
	Master degree	13	4.8
	Others	9	3.3
Occupation	Housewife	174	64.4
	Laborer	15	5.6
	Private sector employee	37	13.7
	Civil servant	44	16.3
Monthly income	IRD 1.000.000 – 5.000.000	116	43
	IRD 6.000.000 – 10.000.000	13	4.8
	IRD 11.000.000 –15.000.000	2	0.7
	IRD >15.000.000	4	1.5
	NA	103	38.1
	Others	32	11.9

### Data collection instruments

The authors developed the questionnaire, which focuses on parents’ roles, by drawing on empirical research concerning parental influence on young learners’ motivation and engagement in tasks ( Butler, 2015a; Tan & Caleon, 2022; Torrecilla & Hernández-Castilla, 2020; Tseng, 2021) to ensure construct validity. This includes: 1) parental involvement (such as assisting with homework, attending school meeting, and actively engaging in their child’s educational journey), 2) parental expectations (setting high academic aspirations and offering encouragement), 3) access to educational resources (such as books, educational technology, and a study-friendly environment), and 4) exposure to enrichment activities (providing diverse experiences like travel, cultural events, and extracurricular programs). We developed the parents’ role questionnaire because the existing questionnaire did not relate to the study’s purposes. Table 2 presents the 19 items of the role of parents. The questionnaire employed a 4-point Likert scale ranging from strongly disagree = 1 to strongly agree = 4.

**Table 2.** Items of the parents’ questionnaire.

Construct	Dimensions	Items
Parents’ role	Parental involvement	1. I ask the teacher regularly about my child’s progress in English.
		2. I help my child with the English tasks at home.
		3. I read English story books with my child.
		4. I attend parental meetings with my child’s English teacher.
	Parental expectation	5. It is important for my child to be good at English.
		6. I expect my child to do well in their English lessons.
		7. I expect my child to get a good grade in English lesson.
		8. I want my child to speak English fluently.
		9. I want my child to enjoy learning English.
	Access to resources	10. I provide English books for my child to learn English at home.
		11. I provide my child with access to the Internet.
		12. I provide access to games in English.
		13. My child has access to English language apps.
		14. My child can watch movies in English at home.
	Enrichment activities	15. My child reads English story books at home.
		16. My child learns English at a language center.
		17. I pay for private tutoring in English.
	Extracurricular activities	18. I encourage my child to participate in activities in English outside school.
		19. I support my child to participate in an English competition such as “spelling bee”.

### Procedure

Ethical approval from the Institutional Review Board (IRB) of the Doctoral School of Education at the University of Szeged (Reference number: 24/2023) was secured prior to data collection. All participants were informed about the study’s objectives, and written consent forms were signed by each prior participating in this survey. The development and validation of the questionnaire concerning the role of parents involved multiple phases: initially, all existing instruments in publications were reviewed; next, items were formulated based on the literature with input from experts. The questionnaire was evaluated by three experts (the co-author and two Indonesian teachers of English). Experts evaluated the content, clarity, and readability of the survey. The questionnaire was clear and covered the content related to the role of parents in motivating young learners to learn English and engage in English tasks. The questionnaire was then compiled and subjected to pilot testing to assess its validity and reliability. The questionnaire was translated to Indonesian language by two experts who fluent English and Indonesian languages and have expertise in teaching English to children. The questionnaire was piloted in November 2024 with parents of the fifth-graders. The online questionnaire in Google Forms was distributed via WhatsApp to the English teachers at primary schools, who forwarded it to parents. Finally, exploratory and confirmatory factor analyses (EFA and CFA) were conducted utilizing the jamovi app (version 2.3.28.0).

## Results

### EFA of the parents’ questionnaire

Before conducting EFA analysis, we ran the KMO of Measurement of Sampling Adequacy (MSA) and Bartlett’s sphericity test to make sure that these data are appropriate for factor analysis. The results showed that the data were appropriate for factor analysis ( Kaiser, 1974) with MSA KMO = .911, and Bartlett’s sphericity test was chi-square = 2534, df = 171, and p = < .001. The fit indices showed that Tucker-Lewis Index (TLI = .976) and Root Mean Square Error of Approximation (RMSEA = .034), indicating a good fit ( Hu & Bentler, 1999). Model test (χ ^2^ = 114, df = 86, p = .025).

Table 3 shows that five factors account for 58.1% of the total variance. Factor 1 (parental expectation) explains 16.03%, while factors 2 (access to resources), 3 (parental involvement), 4 (enrichment activities), and 5 (extracurricular activities) contribute 13.54%, 10.87%, 10.45%, and 7.25%, respectively.

**Table 3.** Result of factor analysis.

Factor	SS Loadings	% of variance	Cumulative %
1	3.05	16.03	16.0
2	2.57	13.54	29.6
3	2.07	10.87	40.4
4	1.99	10.45	50.9
5	1.38	7.25	58.1

**Figure 2.**
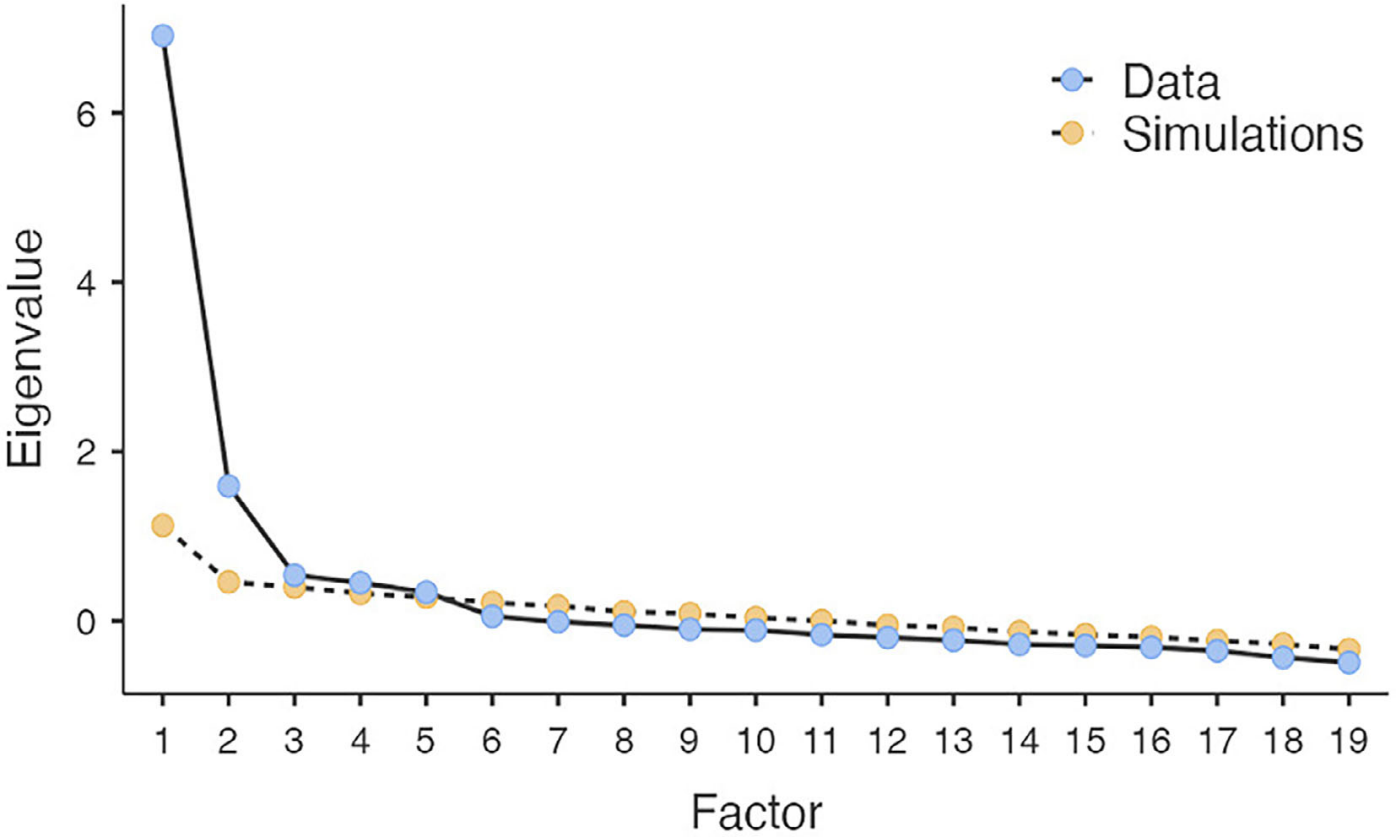
Screen plot of factor analysis.

Figure 2 presents the scree plot supporting the decision on five factors, displaying eigenvalues from a factor analysis. A steep initial decline and subsequent leveling indicate the optimal number of factors to retain, or eigenvalue > 1, due to their significant contribution to variance explanation ( Kaiser, 1974).

Table 4 presents the EFA results, applying the Maximum Likelihood extraction method accompanied by a varimax rotation. The factor loadings span from .496 to .881. Each item’s loadings are classified into five distinct factors: parental involvement, parental expectation, access to resources, enrichment activities, and extracurricular activities. Notably, the strongest indicator of the extracurricular activities construct is the factor loading associated with encouraging children to engage in activities outside of school (item 18, factor loading = .881) ( Hair et al., 2019). Conversely, Item 2 exhibited the weakest loading, signifying a minimal contribution to the parental involvement factor.

**Table 4.** Exploratory factor analysis of the parents’ questionnaire.

	Factor				
Dimensions/items	1	2	3	4	5
**Parental involvement**					
1. I ask the teacher regularly about my child’s progress in English.			.651		
2. I help my child with the English tasks at home.			.496		
3. I read English story books with my child.			.585		
4. I attend parental meetings with my child’s English teacher.			.502		
**Parental expectation**					
5. It is important for my child to be good at English.	.683				
6. I expect my child to do well in their English lessons.	.840				
7. I expect my child to get a good grade in English lesson.	.632				
8. I want my child to speak English fluently.	.761				
9. I want my child to enjoy learning English.	.686				
**Access to resources**					
10. I provide English books for my child to learn English at home.		.514			
11. I provide my child with access to the Internet.		.724			
12. I provide access to games in English.		.660			
13. My child has access to English language apps.		.593			
14. My child can watch movies in English at home		.563			
**Enrichment activities**					
15. My child reads English story books at home.				.566	
16. My child learns English at a language center.				.757	
17. I pay for private tutoring in English.				.617	
**Extracurricular activities**					
18. I encourage my child to participate in activities in English outside school.					.881
19. I support my child to participate in an English competition such as “spelling bee”.					.515

Note. ‘Maximum likelihood’ extraction method was used in combination with a ‘varimax’ rotation.

### CFA of parents’ questionnaire

Table 5 summarizes the findings from a confirmatory factor analysis examining various constructs associated with the influence of parents on students’ motivation and engagement with tasks. Constructs assessed include parental involvement, parental expectation, access to resources, enrichment activities, and extracurricular activities. Each construct is evaluated through multiple items, with factor loadings demonstrating the strength of association between items and their respective constructs. For example, item loadings for parental expectation range from .646 to .869, indicating moderate to strong representation of the construct ( Hair et al., 2019). The Average Variance Extracted (AVE) values span from .41 to .71, highlighting how much variance is captured by the construct in relation to measurement error, with higher values (such as .71 for extracurricular activities) signifying better convergent validity ( Fornell & Larcker, 1981). Reliability is assessed for each construct using Cronbach’s alpha (α) and Composite Reliability (CR), with values above .70 deemed acceptable. Most constructs exhibit strong reliability, such as parental expectation (α = .86, CR = .87) and access to resources (α = .86, CR = .86) ( Tavakol & Dennick, 2011). Even constructs like parental involvement with a slightly lower AVE of .41 have acceptable reliability (α = .73, CR = .74) ( Fornell & Larcker, 1981), indicating that the items reliably measure the underlying concept of each construct. In general, the results indicate that the measurement model is valid and reliable for evaluating the impact of parent-related factors on student motivation and task engagement.

**Table 5.** Factor loadings of constructs.

Constructs	Items	Factor loadings	AVE	α	CR
Parental Involvement	Item1	.713	.41	.73	.74
	Item2	.563			
	Item3	.652			
	Item4	.633			
Parental Expectation	Item5	.714	.57	.86	.87
	Item6	.869			
	Item7	.646			
	Item8	.793			
	Item9	.722			
Access to Resources	Item10	.704	.56	.86	.86
	Item11	.757			
	Item12	.768			
	Item13	.762			
	Item14	.741			
Enrichment Activities	Item15	.777	.46	.80	.80
	Item16	.804			
	Item17	.678			
Extracurricular Activities	Item18	.837	.71	.83	.83
	Item19	.854			

The chi-square statistic (χ ^2^ = 276, df = 142, p < .001) for testing exact model fit reveals a statistically significant outcome, implying an imperfect data-model fit. Nonetheless, given the chi-square test’s sensitivity to large sample sizes, researchers frequently evaluate model adequacy using alternative fit indices. The Comparative Fit Index (CFI = .945) and Tucker-Lewis Index (TLI = .934) both surpass the standard threshold of .90, suggesting an adequate fit ( Hu & Bentler, 1999). Further, the Standardized Root Mean Square Residual (SRMR = .045) and Root Mean Square Error of Approximation (RMSEA = .059) fall within acceptable ranges (SRMR < .08 and RMSEA between .06 and .08), affirming that the model is reasonably well-fitting overall, despite the chi-square result.

Table 6 presents the correlations between five constructs that represent different aspects of how parents influence student motivation and engagement in tasks: parental involvement, parental expectations, access to resources, enrichment activities, and extracurricular activities. The correlations are all positive, indicating that improvement in one area generally corresponds to enhancements in others. Access to resource availability reveals strong correlations with both parental involvement (r = .798) and enrichment activities (r = .756), implying that families with greater resources are more likely to be involved in their child’s education and offer enriching experiences. Additionally, extracurricular activities are strongly associated with access to resources (r = .728) and enrichment activities (r = .705), emphasizing the interrelated nature of these supportive actions. On the other hand, parental expectations show more moderate correlations with the other constructs (ranging from .351 to .531), suggesting that although expectations are linked to other supportive behaviors, they may represent a distinct dimension of parental influence.

**Table 6.** The relationship among the factors of the parents’ role.

	Parental involvement	Parental expectation	Access to resources	Enrichment activities	Extracurricular
Parental involvement	1.000 ^a^				
Parental expectation	.369	1.000 ^a^			
Access to resources	.798	.531	1.000 ^a^		
Enrichment activities	.718	.351	.756	1.000 ^a^	
Extracurricular	.654	.430	.728	.705	1.000 ^a^

**Figure 3.**
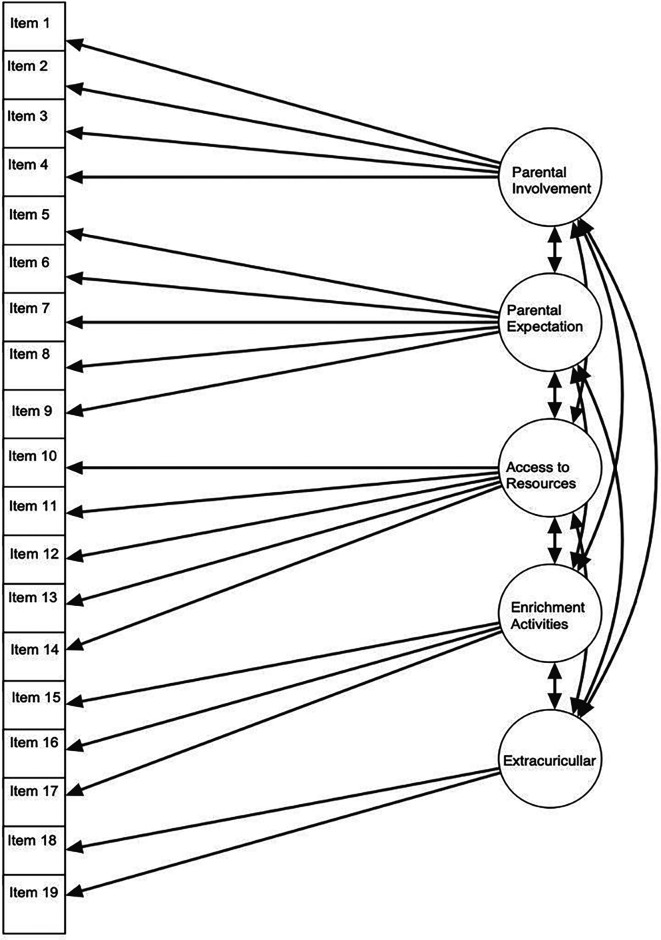
Structure of parents’ role.

Figure 3 illustrates five latent variables or factors: parental involvement, parental expectations, access to resources, enrichment activities, and extracurricular activities. These factors are connected to various observed variables or indicators such as items 1, 2, 3, 4, and 5. The arrows leading from latent variables to observed variables represent factor loadings, indicating the degree to which each indicator supports the underlying construct. For instance, item 6 exhibits a strong association with the factor of parental expectations, possessing a standardized estimate of .869. Similarly, items 18 and 19 show strong correlations with the extracurricular activities factor, with standardized estimates of .837 and .854, respectively. Double-headed arrows connecting multiple latent variables signify relationships among the constructs, such as the strong association between extracurricular activities and access to resources.

Table 7 presents the descriptive statistics for the dimensions of parental roles, indicating generally high levels of agreement across all items, with means fluctuating between 3.06 and 3.73 on a 4-point scale. The highest mean scores were found in parental expectations (M = 3.65 to 3.73), reflecting strong convictions regarding the importance of English and significant aspirations for their children’s English proficiency. This dimension exhibited significant negative skewness and high kurtosis, implying a tendency for responses to cluster toward strong agreement. Parental involvement and access to resources displayed relatively high mean scores (approximately 3.15 to 3.35), accompanied by mild negative skewness and low to moderate kurtosis, indicating slightly skewed but closer to normally distributed responses. Meanwhile, mean scores for enrichment and extracurricular activities were somewhat lower (approximately 3.06 to 3.28), with more even distributions and less skewness, pointing to greater variability in the frequency of these activities. In conclusion, while parental expectations are consistently high, there is more variation in the level of actual involvement in enrichment and extracurricular activities.

**Table 7.** Descriptive statistics of the parents’ role.

Dimension/Items	Mean	SD	Skewness		Kurtosis	
			Statistic	Std. Error	Statistic	Std. Error
**Parental involvement**						
1. I ask the teacher regularly about my child’s progress in English.	3.15	.703	-.221	.148	-.960	.295
2. I help my child with the English tasks at home.	3.30	.665	-.433	.148	-.761	.295
3. I read English story books with my child.	3.17	.764	-.306	.148	-1.227	.295
4. I attend parental meetings with my child’s English teacher.	3.15	.711	-.222	.148	-1.004	.295
**Parental expectation**						
5. It is important for my child to be good at English.	3.65	.570	-1.408	.148	1.005	.295
6. I expect my child to do well in their English lessons.	3.72	.512	-1.625	.148	1.768	.295
7. I expect my child to get a good grade in English lesson.	3.69	.558	-1.626	.148	1.686	.295
8. I want my child to speak English fluently.	3.71	.544	-1.741	.148	2.110	.295
9. I want my child to enjoy learning English.	3.73	.494	-1.563	.148	1.526	.295
**Access to resources**						
10. I provide English books for my child to learn English at home.	3.29	.694	-.457	.148	-.862	.295
11. I provide my child with access to the Internet.	3.35	.667	-.543	.148	-.715	.295
12. I provide access to games in English.	3.22	.681	-.310	.148	-.847	.295
13. My child has access to English language apps.	3.13	.721	-.193	.148	-1.056	.295
14. My child can watch movies in English at home.	3.29	.644	-.353	.148	-.702	.295
**Enrichment activities**						
15. My child reads English story books at home.	3.08	.740	-.125	.148	-1.161	.295
16. My child learns English at a language center.	3.06	.767	-.095	.148	-1.290	.295
17. I pay for private tutoring in English.	3.09	.793	-.153	.148	-1.392	.295
**Extracurricular activities**						
18. I encourage my child to participate in activities in English outside of school.	3.28	.674	-.399	.148	-.803	.295
19. I support my child to participate in an English competition such as “spelling bee”.	3.22	.697	-.333	.148	-.918	.295

## Discussion

Considering EFA and CFA, the questionnaire assessing parents’ roles comprises five components: parental involvement, parental expectations, access to resources, enrichment activities, and extracurricular activities. The CFA results suggest the model fits well, as indicated by fit indices: CFI = .945, TLI = .934, SRMR = .045, RMSEA = .059 ( Hu & Bentler, 1999). Despite some lower figures, average variance extracted (AVE) suggests adequate convergent validity ( Fornell & Larcker, 1981). With Cronbach’s alpha and composite reliability generally over .70 ( Tavakol & Dennick, 2011), the questionnaire demonstrates reliability, especially in the parental expectations construct. This implies a high correlation among the items, effectively capturing their respective factors. Thus, the questionnaire is both valid and reliable for measuring parents’ roles in influencing children’s motivation and task engagement.

## Conclusions

It can be concluded that the questionnaire intended to evaluate parental roles in children’s motivation and task engagement shows robust psychometric qualities in both exploratory and confirmatory factor analyses. The five-factor framework, parental involvement, parental expectations, access to resources, enrichment activities, and extracurricular activities, is validated by satisfactory model fit indices, suggesting a well-fitting measurement model. Strong convergent validity and consistently high reliability measures also confirm that the constructs are assessed both accurately and reliably. These results indicate that the questionnaire serves as a valid and reliable instrument for investigating the impact of different aspects of parental support on children’s motivation and task participation in learning English.

Although the instrument demonstrated both validity and reliability within Indonesian primary schools, its broader applicability may be constrained due to differing parental roles and expectations across various cultural, socio-economic, and educational backgrounds. Additionally, the use of self-reported data introduces the potential for social desirability bias. To enhance its cross-cultural relevance, future research should involve a range of viewpoints and apply the instrument to varied populations.

## Implications

This study has some implications. First, practical implications, this research provides an effective diagnostic tool for teachers, school leaders, and policymakers to pinpoint and understand the particular dimensions of parental involvement that affect student motivation and engagement. By identifying strengths and weaknesses in parental involvement, resources, or expectations, targeted interventions and family engagement initiatives can be designed to improve student success. Schools can create workshops or resources that align with parents’ contributions to supporting their children’s English learning.

Pedagogically, the study highlights the critical role of recognising parents as active collaborators in the educational journey. Educators can modify communication methods and classroom activities to better align with the most influential types of parental support, such as promoting enrichment activities or engaging parents in extracurricular events. Additionally, awareness of the differing parental expectations can help educators improve student learning experiences, fostering motivation and engagement.

Theoretically, the study improves the theoretical understanding of the impact of parental behaviour in educational psychology and motivation studies. By supporting a multidimensional view of parental roles, it underscores that family support is diverse, consisting of separate yet interconnected aspects. The findings are consistent with self-determination theory, providing a more nuanced perspective to explore how different forms of parental participation influence student motivation and task engagement in learning English.

## Ethical approval

We obtained ethical approval from The Doctoral School of Education’s Institutional Review Board at the University of Szeged (Reference number: 24/2023).

## Informed consent

All adult participants gave written informed and voluntary consent.

## Consent for publication

This manuscript is unpublished and not under review by another journal.

## Data Availability

The data cannot be shared publicly due to restrictions for ethical and security reasons. The IRB of the doctoral school of education at the University of Szeged did not allow us to share our data with a third party. The datasets from this study can be requested from the corresponding author at maisrilena@fip.unp.ac.id Figshare: *Parents’ roles in young learners’ motivation and task engagement in Indonesian primary schools*: *Questionnaire development and validation.* https://doi.org/10.6084/m9.figshare.30302089 ( Lena, 2025a). The project contains the following extended data:
- Parents’ questionnaire Data are available under the terms of the CC BY 4.0 Figshare: *Parents’ roles in young learners’ motivation and task engagement in Indonesian primary schools*: *Questionnaire development and validation.* https://doi.org/10.6084/m9.figshare.30302086 ( Lena, 2025b). The project contains the following extended data:
- Consent form for participation Data are available under the terms of the CC BY 4.0
