# Parents’ roles in young learners’ motivation and task engagement in Indonesian primary schools: Questionnaire development and validation

**DOI:** 10.12688/f1000research.170391.2

**Published:** 2025-12-24

**Authors:** Mai Sri Lena, Marianne Nikolov

**Affiliations:** 1 Doctoral school of education, University of Szeged, Szeged, H-6720, Hungary; 2 Department of Elementary School Teacher Education, Universitas Negeri Padang, Padang, 25171, Indonesia; 3 Institute of English Studies, University of Pécs, Pécs, H-7622, Hungary

**Keywords:** Parents’ role, young learners, motivation, task engagement, primary school, validation

## Abstract

**Background:**

Parental involvement in a child’s second language learning is important because it affects the process and outcomes. Limited research has been conducted in the Indonesian context on what roles parents play in their children’s motivation and task engagement. Therefore, the study aims to develop, pilot, and validate a questionnaire on the roles of parents in motivating and engaging young learners with English tasks assigned by teachers.

**Methods:**

This study was quantitative in nature. Participants were 270 parents of fifth graders learning English at nine public and private schools in Padang. The instrument was developed by analysing the literature and existing tools and creating new items. After getting expert feedback and piloting the survey, we assessed its validity and reliability. Research questions examined factors affecting its effectiveness.

**Results:**

The questionnaire was analysed through EFA and CFA via Jamovi. EFA identified five dimensions: (1) parental involvement, (2) expectations, (3) access to resources, (4) enrichment and (5) extracurricular activities. The CFA fit indices (CFI = .945, TLI = .934, SRMR = .045, RMSEA = .059) confirmed the model’s suitability. The questionnaire showed strong validity and reliability, with measures exceeding.70, making it effective for gathering data on parental roles in Indonesian children’s learning of English.

**Conclusions:**

This validation study offers an effective diagnostic tool for teachers, administrators, and policymakers to pinpoint the particular dimensions of parental involvement that affect children’s motivation and task engagement as they learn English. The findings highlight the critical roles of recognising parents as active collaborators along the language learning journey. The study adds to the theoretical understanding of the impact of parental behaviour in educational psychology and motivation studies. The findings are consistent with self-determination theory, providing a more nuanced perspective to explore how different forms of parental participation influence student motivation and task engagement in learning English.

## Introduction

Young learners’ (YLs) motivation to learn English is influenced by internal and external factors. These include their interest in English, interactions with teachers, peers, and parents, the classroom environment, and the societal value of English. In Indonesia, where English is a foreign language, it is crucial for YLs to enjoy learning and use all learning opportunities despite limited exposure outside of school. Indonesian children often start learning English early, making it vital to nurture motivation for long-term language development.

Understanding how children’s motivation develops is key to forming effective teaching methods and addressing challenges such as unequal access to instruction and varying levels of parental involvement. Research shows that motivation significantly affects learners’ engagement, persistence, and achievement in language learning ( Lamb, 2017), making it essential for improving English education in Indonesia.

Furthermore, according to the Ministry of Education and Culture Regulation Number 51 of 2018 on the admission of new students to primary schools in Indonesia, YLs are 7-12-year-old pupils in primary schools ( Minister of Education and Culture, 2018). Given English’s status as a global language, introducing it to young learners at an early stage is seen as advantageous for their exposure to the language. Therefore, English is a compulsory subject for students in elementary schools starting from grade three, based on Ministerial Regulation Number 12 of 2024 concerning the curriculum in early childhood education, elementary and secondary education ( Minister of Education Culture Research and Technology, 2024). Some schools provide English as a subject from grade one to six, and some others from grade three to six, depending on the school’s decision. However, little research has been conducted on YLs’ needs, who and what motivates them to learn English at the primary school level. Therefore, there is a need to investigate this topic further.

Multiple publications revealed that parental involvement in a child’s second language learning is important because it affects children’s motivation (e.g., Choi et al., 2024; Liang et al., 2024; Mihaljević Djigunović & Nikolov, 2019) and engagement (e.g., Fan & Williams, 2010; Liang et al., 2024). However, no empirical studies have explored how parents perceive their roles in their children’s learning of English at the primary school level. Previously used questionnaires focus on students ( Butler & Le, 2018; Liu et al., 2016; Oga-Baldwin et al., 2017; Wang & Bai, 2023) and teachers ( Kalayci & Ergül, 2020) and were designed for Western contexts, making them less suitable for use with Indonesian parents of young learners.

Therefore, this research introduces a novel approach by bridging a contextual and methodological gap, presenting the first validated survey tailored to capture Indonesian parents’ views on their roles in enhancing their children’s motivation and task engagement in learning English. This research, by anchoring the instrument in the specific educational and cultural context, delivers unique data at a crucial moment—right before English is mandated as a core subject from an early age in all Indonesian primary schools. The results aim to academically augment motivation studies in less-explored settings and to practically guide improvements in English education by promoting greater family participation, especially the involvement of parents.

This study aimed to answer two research questions:

1. What underlying factors structure the questionnaire measuring parents’ roles in their children’s English learning motivation and task engagement?
2. How valid and reliable is the questionnaire piloted with parents of fifth-grade learners of English?

Before designing the questionnaire, we reviewed the literature. In the first section, we explain how we decided which working definitions to use for various constructs related to how parents impact their children’s English language learning motivation and task engagement, how these were researched, and what the main findings were. Then, we outline the key variables we assumed to impact Indonesian young learners’ motivation and engagement, how we developed a survey for their parents, and how the data collection instrument worked with 270 participants in a pilot study.

## Literature review

### Parents’ roles in English language learning

Parental involvement is crucial in children’s English learning, influencing motivation and engagement ( Liang et al., 2024) and enhancing academic interest and passion, study habits, and grades ( Shebani et al., 2025). Research indicates that parental socioeconomic status (SES) combind as their education, income, and occupation ( Ma et al., 2023; Sirin, 2005) positively correlates with L2 achievements ( Ma et al., 2023), academic results ( Balsa et al., 2018), and English learning ability ( Butler & Le, 2018).

University-educated mothers positively influenced their children’s English proficiency ( Jalili, 2017; Tan & Caleon, 2022). Low parental education correlated with higher primary education drop-out rates in D.G.Khan ( Ahsan et al., 2015). Family income affected children’s English learning engagement in elementary schools Hong Kong: low-income children were less engaged than those from middle-income families ( Poon, 2020).

Various research papers have assessed the role of parental involvement in enhancing children’s academic achievements. The engagement of Chinese parents in their children’s English studies shows a significant link to the children’s proficiency in the target language ( Butler & Le, 2018). Support provided by parents at home plays a crucial role in boosting students’ academic performance ( Tan & Caleon, 2022; Torrecilla & Hernández-Castilla, 2020). According to Kim et al. (2020), the psychological and behavioral participation of parents has a more profound effect on the motivation and accomplishments of their children than the parents’ educational background. Communication between parents and teachers has also been shown to augment students’ achievements ( Tan & Caleon, 2022) as well as their engagement and motivation to master English ( Fan & Williams, 2010). Involvement of parents in extracurricular activities and attendance at parent meetings has a notable influence on student performance in elementary schools in Latin America ( Torrecilla & Hernández-Castilla, 2020).

**Figure 1.**
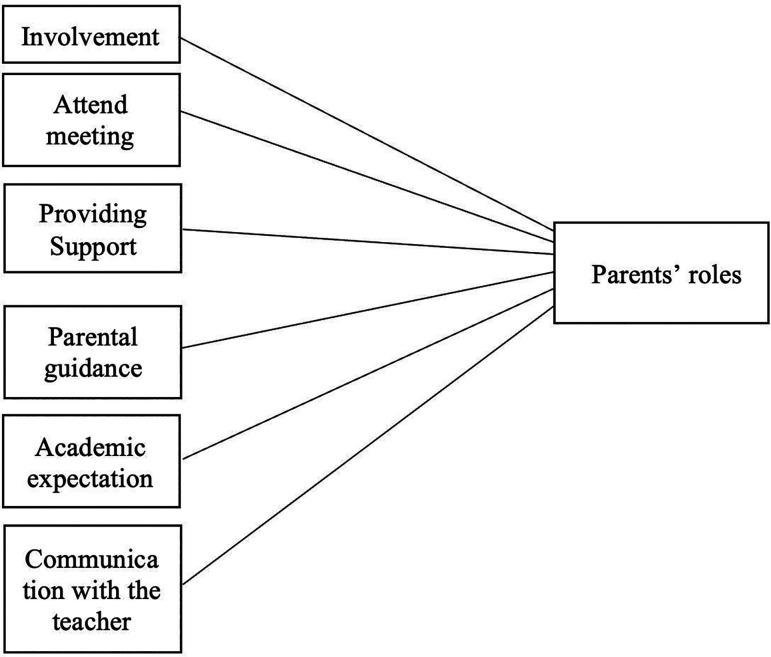
Parents’ roles construct.

Additionally, parental guidance is a positive predictor of students’ intrinsic motivation to advance their English skills ( Fan & Williams, 2010), while parents’ academic expectations also align positively with students’ English proficiency ( Butler & Le, 2018), achievements ( Tan & Caleon, 2022), engagement levels, and intrinsic motivation in English ( Fan & Williams, 2010). Figure 1 illustrates some parents’ roles in young learners’ English learning based on the literature.

### Motivation and task engagement in EFL contexts

Motivation is identified as a complex entity, consisting of three components: the aspiration to learn the language, perceptions regarding language learning, and the intensity of motivation demonstrated by continued effort in language acquisition ( Gardner, 2010). In English language learning, especially at the primary school level, motivation has been shown to influence learners’ persistence, participation, and language outcomes ( Dörnyei & Ushioda, 2011). Motivation plays a pivotal role in language learning; it determines the rate and success of acquiring a new language ( Gardner, 1985). This study conceptualizes motivation as the learners’ willingness, interest, and enthusiasm toward learning English. It is based on self-determination in which motivation is divided into two types: intrinsic and extrinsic motivation ( Deci & Ryan, 1985; Ryan & Deci, 2020). **Intrinsic motivation** is defined as the interest or enjoyment in English tasks, while **extrinsic motivation** is encouraged by external rewards, pressures, or outcomes. These types of motivation are widely used in educational psychology and are appropriate for young learners’ development.

Task engagement refers to students’ involvement in completing tasks during the process of language learning ( Philp & Duchesne, 2016). It is understood as a construct with four distinct dimensions: behavioral, cognitive, emotional, and social engagement ( Philp & Duchesne, 2016). Behavioral engagement involves learners actively participating in tasks ( Philp & Duchesne, 2016), which can be assessed through factors like time dedicated to tasks and responding to questions ( Dörnyei & Kormos, 2000; Oga-Baldwin, 2019). Emotional engagement encompasses learners’ emotional connections and reactions to class activities ( Sang & Hiver, 2021). Cognitive engagement relates to alertness, continuous attention, active mental processes, mental effort, and self-regulation techniques ( Dao, 2021). Agentic engagement is defined by learners’ actions aimed at influencing classroom instruction ( Oga-Baldwin, 2019). Social engagement refers to the extent to which a student follows written and unwritten classroom rules of behavior, for example, coming to school and class on time, interacting appropriately with teachers and peers ( Finn & Zimmer, 2012).

Parents play a significant role in motivating young learners to learn English and in their engagement with tasks. Research indicates that parental involvement has a notable effect on children’s motivation ( Choi et al., 2024; Liang et al., 2024; Sumanti & Muljani, 2021; Tong et al., 2021) and engagement ( Liang et al., 2024). Interaction between parents and teachers is shown to enhance students’ motivation and engagement in English learning ( Finn & Zimmer, 2012).

### Parental questionnaires on young learners’ motivation and task engagement

Self-report questionnaires are the most frequently used tools to assess motivation and engagement in tasks. Some prior studies developed and adapted questionnaires to investigate motivation and engagement in tasks. For instance, Butler (2015b) and Butler and Le (2018) in China used parental a survey comprising various aspects related to their children’s learning of the English language. They included: (a) the socioeconomic status (SES) of parents, indicated by household income and level of education; (b) indirect actions by parents aimed at supporting English learning, for instance, fostering a literacy-rich home environment and using English at part of their jobs; (c) direct parental actions to facilitate English education, for example, engaging in their children’s study process and arranging for private English tutoring post-school hours; (d) parental beliefs regarding English education, including the significance of English; and (e) specific beliefs parents have about their own child, like their expectation concerning the child’s success in learning English. Butler (2015b) found that higher SES parents adjusted their parenting to children’s changing needs and, through their ability to provide more frequent English use outside the classroom, increasingly fostered their children’s competence and self-determined motivation. In contrast, lower SES parents tended to remain controlling and often failed to cultivate such competence and self-determined motivation. Butler and Le (2018) revealed strong correlations between SES and parents’ views on English, parenting styles, number of Chinese books at home, involvement in children’s English learning, and beliefs and expectations about their children’s English ability. Parents’ autonomous (rather than controlled) parenting, beliefs, and expectations all positively related to their children’s English achievement.

Torrecilla and Hernández-Castilla (2020) in Latin America used a parental survey on household assets, their connection with the student and school, and their general contentment with the school. They found that parents’ engagement in school and learning directly improved YLs’ academic performance. Third-grade students whose parents helped with homework, especially when the mother provided support, achieved higher results, and parents’ attendance at meetings with the principal and teachers, as well as participation in extracurricular activities, also positively affected achievements.

Wang et al. (2023) used the Chinese version of a parental involvement scale from a previous study covering three aspects: personal, cognitive, and behavioral involvement. Personal involvement measured perceived care about school life and emotions (e.g., easing emotions during learning difficulties). Cognitive involvement assessed exposure to stimulating activities and materials (e.g., buying study materials). Behavioral involvement evaluated parents’ participation in school activities (e.g., controlling online time). Their study showed that parents’ involvement directly increased children’s learning engagement and indirectly did so through children’s perceptions of that involvement, with learning engagement mediating the link between parental involvement and academic achievement. Parental involvement significantly predicted children’s English performance through its effect on engagement.

However, no research has been conducted in the Indonesian context on the development and validation of instruments to measure parents’ involvement in their children’s motivation and engagement with English tasks. Therefore, it is necessary to develop and validate a questionnaire on young learners’ motivation and task engagement from the perspectives of their parents. By doing so, we aim to enhance local educational practices and add to the existing body of knowledge in English language learning research in a new context.

## Method

### Research design

The study was quantitative in nature. It involved the measurement and analysis of numerical data ( Creswell & Creswell, 2018). Its focus was on the development and validation of a new instrument in primary schools in Indonesia.

### Context of the study

The research was carried out in Padang elementary schools, where English is taught as a foreign language and is a required subject for YLs at elementary school from grade three. Children in both private and public elementary schools learn English for 70 minutes a week with English teachers who have an English educational background.

### Participants

Participants were a large sample parents of fifth graders. We used a convenience sampling technique in selecting them, as this offers quick access to willing participants, enabling fast, efficient data collection while reducing financial and logistical costs ( Cohen et al., 2018). It is especially useful for testing instruments and convenience sampling allows researchers to collect data in naturalistic settings. However, we acknowledge that convenience sampling may limit the generalizability of the findings to other populations, but this was the feasible way forward.

The study involved 270 parents of fifth-grade students, and their characteristics can be found in Table 1. Most respondents were female (88.5%), with 48.9% aged between 38 and 44. Many parents (46.3%) reported their English proficiency to be at the intermediate level, and every fifth respondent used English regularly. They used it primarily for entertainment purposes (46.96%). More than half of the participants had high-school education (60.4%) and were housewives (64.4%). Furthermore, 43% of respondents had a monthly income between IDR 1.000.000 and 5.000.000, while 38.1% chose not to disclose their incomes.

**Table 1.** Description of participants in this study.

Personal information	Category	Frequency	Percentage (%)
Gender	Male	31	11.5
	Female	239	88.5
Age	31-37	78	37.1
	38-44	132	48.9
	45-51	48	17.8
	52-58	8	3
	59-65	4	1.5
English proficiency	Low	64	23.7
	Beginner-intermediate	73	37
	Intermediate	125	46.3
	Advanced	8	3
Using English	Regularly	58	21.5
	Sometimes	107	39.6
	Rarely	101	37.4
	Never	4	1.5
What for	Use it in my job	13	19.69
	Use it for entertainment	31	46.96
	Use it for my interest	12	18.18
	Use it for travel	10	15.15
Education background	High school	163	60.4
	Diploma degree	25	9.3
	Bachelor degree	60	22.2
	Master degree	13	4.8
	Others	9	3.3
Occupation	Housewife	174	64.4
	Laborer	15	5.6
	Private sector employee	37	13.7
	Civil servant	44	16.3
Monthly income	IRD 1.000.000 – 5.000.000	116	43
	IRD 6.000.000 – 10.000.000	13	4.8
	IRD 11.000.000 –15.000.000	2	0.7
	IRD >15.000.000	4	1.5
	NA	103	38.1
	Others	32	11.9

### Data collection instrument

A questionnaire was developed for gathering data, as previously used instruments did not meet the purpose of the present study. It focused on parents’ roles by drawing on empirical research concerning parental influence on YLs’ motivation and engagement in tasks ( Butler, 2015a; Tan & Caleon, 2022; Torrecilla & Hernández-Castilla, 2020; Tseng, 2021) to ensure construct validity. The instrument included five dimensions: 1) parental involvement (e.g., assisting with homework, attending school meeting, and actively engaging in their child’s educational journey), 2) parental expectations (e.g., setting high academic aspirations, offering encouragement), 3) access to educational resources (such as books, educational technology, and a study-friendly environment), and 4) exposure to enrichment activities (e.g., providing diverse experiences like travel, cultural events, and 5) access to extracurricular programs. Table 2 presents the 19 items on the roles of parents. We used a 4-point Likert scale ranging from strongly disagree = 1 to strongly agree = 4, to avoid a middle choice and to make sure respondents took sides.

**Table 2.** Items of the parents’ questionnaire.

Construct	Dimensions	Items
Parents’ roles	Parental involvement	1. I ask the teacher regularly about my child’s progress in English.
		2. I help my child with the English tasks at home.
		3. I read English story books with my child.
		4. I attend parental meetings with my child’s English teacher.
	Parental expectations	5. It is important for my child to be good at English.
		6. I expect my child to do well in their English lessons.
		7. I expect my child to get a good grade in English lesson.
		8. I want my child to speak English fluently.
		9. I want my child to enjoy learning English.
	Access to resources	10. I provide English books for my child to learn English at home.
		11. I provide my child with access to the Internet.
		12. I provide access to games in English.
		13. My child has access to English language apps.
		14. My child can watch movies in English at home.
	Enrichment activities	15. My child reads English story books at home.
		16. My child learns English at a language center.
		17. I pay for private tutoring in English.
	Extracurricular activities	18. I encourage my child to participate in activities in English outside school.
		19. I support my child to participate in an English competition such as “spelling bee”.

### Procedure

Ethical approval from the Institutional Review Board (IRB) of the Doctoral School of Education at the University of Szeged (Reference number: 24/2023) was secured prior to data collection. All participants were informed about the study’s objectives, and written consent forms were signed by all parents prior to participating in this survey.

The development and validation of the questionnaire concerning the roles of parents involved multiple phases: initially, all existing instruments in publications were reviewed; next, items were formulated based on the literature with input from experts. The questionnaire was evaluated by three experts (the co-author and two Indonesian teachers of English). Experts evaluated the content, clarity, and readability of the survey. They agreed that the questionnaire was clear and covered the content related to the roles of parents in motivating young learners to learn English and engage in English tasks.

Then the instrument was compiled and subjected to pilot testing to assess its validity and reliability. The text was translated to Indonesian language by two bilingual experts who were fluent in the English and Indonesian languages and had expertise in teaching English to children. Piloting took place in November 2024 with parents of fifth graders. The online questionnaire in Google Forms was distributed via WhatsApp to the English teachers at primary schools, who forwarded it to parents.

### Data analysis

Exploratory and confirmatory factor analyses (EFA and CFA) were conducted with the help of the Jamovi app (version 2.3.28.0; The Jamovi project, 2022). This tool assists in performing complex data analyses and deriving meaningful conclusions from the collected data. Before the main analysis, we conducted descriptive analysis.

Table 3 presents the descriptive statistics for the dimensions of parental roles: they indicate generally high levels of agreement across all items between 3.06 and 3.73 on a 4-point scale. The highest mean scores were found in parental expectations (M = 3.65 to 3.73), reflecting strong convictions regarding the importance of English and parents’ significant aspirations for their children’s English proficiency. This dimension exhibited significant negative skewness and high kurtosis, implying a tendency for responses to cluster toward strong agreement. Parental involvement and access to resources displayed relatively high mean scores (between 3.15 to 3.35), accompanied by mild negative skewness and low to moderate kurtosis, indicating slightly skewed but closer to normally distributed responses. Mean scores for enrichment and extracurricular activities were somewhat lower (between 3.06 to 3.28), with more even distributions and less skewness, pointing to greater variability in the frequency of these activities. In conclusion, while parental expectations are consistently high, there is more variation in the level of actual involvement in enrichment and extracurricular activities.

**Table 3.** Descriptive statistics of the parents’ roles.

Dimension/Items	Mean	SD	Skewness		Kurtosis	
			Statistic	Std. Error	Statistic	Std. Error
**Parental involvement**						
1. I ask the teacher regularly about my child’s progress in English.	3.15	.703	-.221	.148	-.960	.295
2. I help my child with the English tasks at home.	3.30	.665	-.433	.148	-.761	.295
3. I read English story books with my child.	3.17	.764	-.306	.148	-1.227	.295
4. I attend parental meetings with my child’s English teacher.	3.15	.711	-.222	.148	-1.004	.295
**Parental expectation**						
5. It is important for my child to be good at English.	3.65	.570	-1.408	.148	1.005	.295
6. I expect my child to do well in their English lessons.	3.72	.512	-1.625	.148	1.768	.295
7. I expect my child to get a good grade in English lesson.	3.69	.558	-1.626	.148	1.686	.295
8. I want my child to speak English fluently.	3.71	.544	-1.741	.148	2.110	.295
9. I want my child to enjoy learning English.	3.73	.494	-1.563	.148	1.526	.295
**Access to resources**						
10. I provide English books for my child to learn English at home.	3.29	.694	-.457	.148	-.862	.295
11. I provide my child with access to the Internet.	3.35	.667	-.543	.148	-.715	.295
12. I provide access to games in English.	3.22	.681	-.310	.148	-.847	.295
13. My child has access to English language apps.	3.13	.721	-.193	.148	-1.056	.295
14. My child can watch movies in English at home.	3.29	.644	-.353	.148	-.702	.295
**Enrichment activities**						
15. My child reads English story books at home.	3.08	.740	-.125	.148	-1.161	.295
16. My child learns English at a language center.	3.06	.767	-.095	.148	-1.290	.295
17. I pay for private tutoring in English.	3.09	.793	-.153	.148	-1.392	.295
**Extracurricular activities**						
18. I encourage my child to participate in activities in English outside of school.	3.28	.674	-.399	.148	-.803	.295
19. I support my child to participate in an English competition such as “spelling bee”.	3.22	.697	-.333	.148	-.918	.295

## Results

To address the research question regarding the underlying factors that structure the questionnaire measuring parents’ roles in their children’s English learning motivation and task engagement, in this section we present the results of EFA and CFA. Furthermore, we present the reliability, convergent, and divergent validity of the questionnaire.

### EFA of the parents’ questionnaire

Before conducting EFA analysis, we ran the KMO of Measurement of Sampling Adequacy (MSA) and Bartlett’s sphericity test to make sure that these data are appropriate for factor analysis. The results showed that the data were appropriate for factor analysis ( Kaiser, 1974) with MSA KMO = .911, and Bartlett’s sphericity test was chi-square = 2534, df = 171, and p = < .001. The fit indices showed that Tucker-Lewis Index (TLI = .976) and Root Mean Square Error of Approximation (RMSEA = .034), indicating a good fit ( Hu & Bentler, 1999). Model test (χ ^2^ = 114, df = 86, p = .025).

The result of the EFA showed that there were five factors in the parents’ questionnaire, along the five dimensions. The five factors accounted for 58.1% of the total variance. Factor 1 (parental expectation) explains 16.03%, while factors 2 (access to resources), 3 (parental involvement), 4 (enrichment activities), and 5 (extracurricular activities) contribute 13.54%, 10.87%, 10.45%, and 7.25%, respectively. See details in Table 4.

**Table 4.** Result of factor analysis.

Factor	SS Loadings	% of variance	Cumulative %
1	3.05	16.03	16.0
2	2.57	13.54	29.6
3	2.07	10.87	40.4
4	1.99	10.45	50.9
5	1.38	7.25	58.1

**Figure 2.**
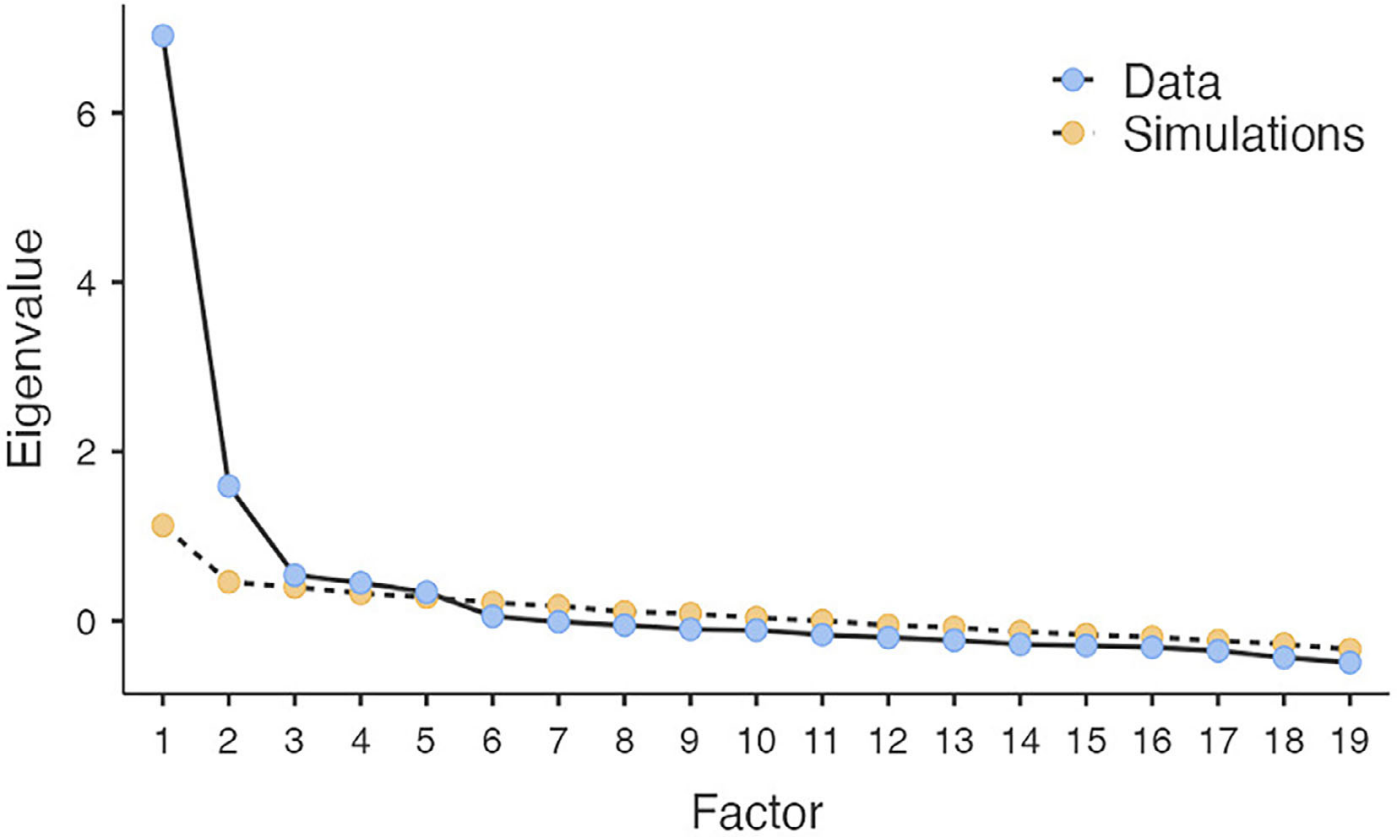
Screen plot of factor analysis.

Figure 2 presents the scree plot supporting the decision on five factors, displaying eigenvalues from a factor analysis. A steep initial decline and subsequent leveling indicate the optimal number of factors to retain, or eigenvalue > 1, due to their significant contribution to variance explanation ( Kaiser, 1974).

When conducting the EFA, we applied the Maximum Likelihood extraction method accompanied by a varimax rotation. The factor loadings span from.496 to.881. Each item’s loadings are classified into five distinct factors: parental involvement, parental expectation, access to resources, enrichment activities, and extracurricular activities. Parental involvement encompassed direct support behaviors, including, for example, helping children with the English tasks and communicating with the English teachers about the child’s progress. Item 2 exhibited the weakest loading, signifying a minimal contribution to the parental involvement factor. Parental expectations represented the aspirations and academic performance levels that parents set for their children, for example, expecting the child to do well in English and to speak English fluently. Access to resources included access to learning materials and tools at home, such as English books, the internet, games, and English language apps. Enrichment activities reflected parents’ efforts to broaden their children’s learning through non-formal learning opportunities at English language centres or with private tutors. Extracurricular activities were organized skill-building programs after typical school hours. Notably, the strongest indicator of the extracurricular activities construct is the factor loading associated with encouraging children to engage in activities outside of school (item 18, factor loading = .881) ( Hair et al., 2019). Together, these factors demonstrate the multifaceted nature of parental engagement in children’s English learning environments. Table 5 presents the EFA results.

**Table 5.** Exploratory factor analysis of the parents’ questionnaire.

	Factor				
Dimensions/items	1	2	3	4	5
**Parental involvement**					
1. I ask the teacher regularly about my child’s progress in English.			.651		
2. I help my child with the English tasks at home.			.496		
3. I read English story books with my child.			.585		
4. I attend parental meetings with my child’s English teacher.			.502		
**Parental expectation**					
5. It is important for my child to be good at English.	.683				
6. I expect my child to do well in their English lessons.	.840				
7. I expect my child to get a good grade in English lesson.	.632				
8. I want my child to speak English fluently.	.761				
9. I want my child to enjoy learning English.	.686				
**Access to resources**					
10. I provide English books for my child to learn English at home.		.514			
11. I provide my child with access to the Internet.		.724			
12. I provide access to games in English.		.660			
13. My child has access to English language apps.		.593			
14. My child can watch movies in English at home		.563			
**Enrichment activities**					
15. My child reads English story books at home.				.566	
16. My child learns English at a language center.				.757	
17. I pay for private tutoring in English.				.617	
**Extracurricular activities**					
18. I encourage my child to participate in activities in English outside school.					.881
19. I support my child to participate in an English competition such as “spelling bee”.					.515

Note. ‘Maximum likelihood’ extraction method was used in combination with a ‘varimax’ rotation.

### CFA of the parents’ questionnaire

A confirmatory factor analysis examined various constructs associated with the parental influence on students’ motivation and engagement with tasks. Constructs assessed included the same five dimension in the EFA. Each construct was evaluated through multiple items, with factor loadings demonstrating the strength of association between items and their respective constructs. For example, item loadings for parental expectation ranged from .646 to .869, indicating its moderate to strong representation in the construct ( Hair et al., 2019). The Average Variance Extracted (AVE) values span from .41 to .71, highlighting how much variance is captured by the construct in relation to measurement error, with higher values (such as .71 for extracurricular activities) signifying better convergent validity ( Fornell & Larcker, 1981). Reliability is assessed for each construct using Cronbach’s alpha (α) and Composite Reliability (CR), with values above .70 deemed acceptable. Most constructs exhibit strong reliability, such as parental expectation (α = .86, CR = .87) and access to resources (α = .86, CR = .86) ( Tavakol & Dennick, 2011). Even a construct like parental involvement with a slightly lower AVE of .41 had acceptable reliability (α = .73, CR = .74) ( Fornell & Larcker, 1981), indicating that the items reliably measure the underlying concept of each construct.

**Table 6.** Factor loadings of constructs.

Constructs	Items	Factor loadings	AVE	α	CR
Parental Involvement	Item1	.713	.41	.73	.74
	Item2	.563			
	Item3	.652			
	Item4	.633			
Parental Expectation	Item5	.714	.57	.86	.87
	Item6	.869			
	Item7	.646			
	Item8	.793			
	Item9	.722			
Access to Resources	Item10	.704	.56	.86	.86
	Item11	.757			
	Item12	.768			
	Item13	.762			
	Item14	.741			
Enrichment Activities	Item15	.777	.57	.80	.80
	Item16	.804			
	Item17	.678			
Extracurricular Activities	Item18	.837	.71	.83	.83
	Item19	.854			

Overall, the results indicate that the measurement model is valid and reliable for evaluating the impact of parent-related factors on their children’s motivation and task engagement. Table 6 provides factor loadings of the five dimension constructs.

The chi-square statistic (χ ^2^ = 276, df = 142, p < .001) for testing exact model fit reveals a statistically significant outcome, implying an imperfect data-model fit. Nonetheless, given the chi-square test’s sensitivity to large sample sizes, researchers frequently evaluate model adequacy using alternative fit indices. The Comparative Fit Index (CFI = .945) and TLI = .934 both surpass the standard threshold of .90, suggesting an adequate fit ( Hu & Bentler, 1999). Furthermore, the Standardized Root Mean Square Residual (SRMR = .045) and RMSEA = .059 fall within acceptable ranges (SRMR < .08 and RMSEA between .06 and .08), affirming that the model is reasonably well-fitting overall, despite the chi-square result.

The correlations among the five dimensions represent different aspects of support (parental involvement, parental expectations, access to resources, enrichment activities, and extracurricular activities) were all positive. This result indicates that improvement in one area generally corresponds to enhancements in others. Access to resource availability reveals strong correlations with both parental involvement (r = .798) and enrichment activities (r = .756), implying that families with greater resources are more likely to be involved in their child’s education and they tend to offer more enriching experiences. Additionally, extracurricular activities are strongly associated with access to resources (r = .728) and enrichment activities (r = .705), again emphasizing the interrelated nature of these supportive actions. However, parental expectations show more moderate correlations with the other constructs (ranging from .351 to .531), suggesting that although expectations are linked to other supportive behaviors, they may represent a distinct dimension of parental influence. Table 7 presents the relationships among these factors reflecting the parents’ roles. The √AVE for each factor, show satisfactory convergent validity, as all five √AVE values on the diagonal exceed the .50 cutoff. However, several inter-factor correlations involving parental involvement, access to resources, and enrichment activities exceed their respective √AVE values, partially violating the Fornell–Larcker criterion and weakening discriminant validity. Thus, although each factor is measured reliably, some constructs may not be fully distinct. Parental involvement overlaps conceptually with access to resources and enrichment activities, suggesting that these dimensions may be closely related facets of parental support for children’s learning rather than clearly separate constructs.

**Table 7.** The relationship among the factors of the parents’ roles.

	Parental involvement	Parental expectation	Access to resources	Enrichment activities	Extracurricular
Parental involvement	.643				
Parental expectation	.369	.755			
Access to resources	.798	.531	.748		
Enrichment activities	.718	.351	.756	755	
Extracurricular	.654	.430	.728	.705	.849

Furthermore, we evaluated discriminant validity using the heterotrait–monotrait (HTMT) ratio of correlations. The HTMT values ranged from .377 to .748, remaining comfortably below both the conservative .85 threshold and the more liberal .90 cut-off ( Henseler et al., 2015). These findings suggest that the constructs have adequate discriminant validity and are statistically distinguishable from each other. Table 8 presents the HTMT ratio of correlations.

**Table 8.** The HTMT ratio of correlations.

	Parental involvement	Parental expectation	Access to resources	Enrichment activities	Extracurricular
Parental involvement	1.000	.404	.733	.733	.663
Parental expectation	.404	1.000	.538	.377	.432
Access to resources	.733	.538	1.000	.748	.732
Enrichment activities	.733	.377	.748	1.000	.705
Extracurricular	.663	.404	.732	.705	1.000

**Figure 3.**
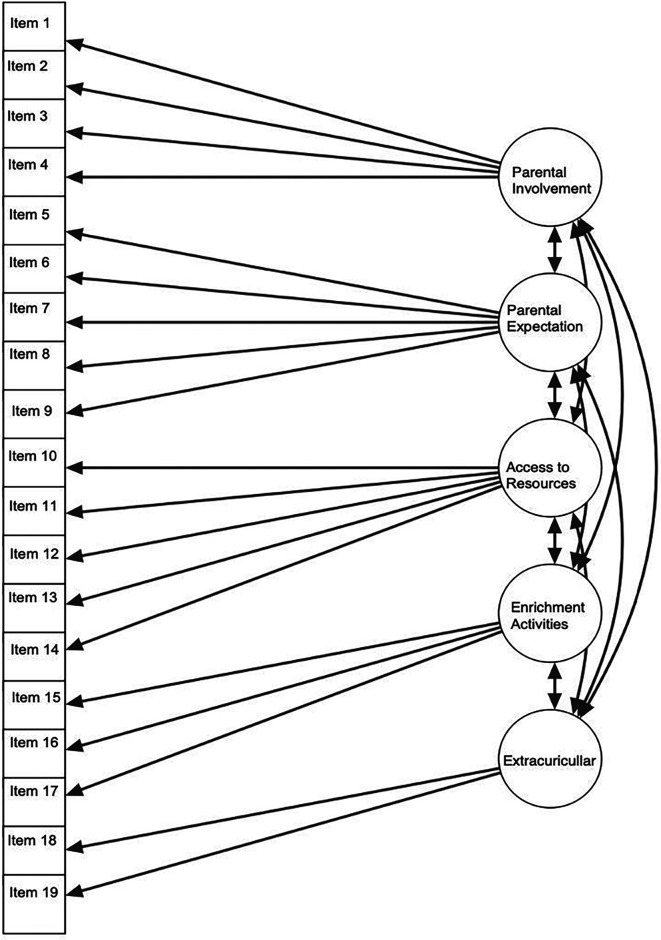
Structure of parents’ roles.

Figure 3 illustrates the five latent variables as factors: parental involvement, parental expectations, access to resources, enrichment activities, and extracurricular activities. These factors are connected to various observed variables or indicators such as items 1, 2, 3, 4, and 5. The arrows leading from latent variables to observed variables represent factor loadings, indicating the degree to which each indicator supports the underlying construct. For instance, item 6 exhibits a strong association with the factor of parental expectations, possessing a standardized estimate of .869. Similarly, items 18 and 19 show strong correlations with the extracurricular activities factor, with standardized estimates of .837 and .854, respectively. Double-headed arrows connecting the five multiple latent variables signify relationships among the constructs, for example, showing the strong association between extracurricular activities and access to resources.

## Discussion

Considering EFA and CFA, the questionnaire assessing parents’ roles comprises five components: parental involvement, parental expectations, access to resources, enrichment activities, and extracurricular activities. These five factors exhibit a strong correspondence with theoretical foundations that shape children’s intrinsic and extrinsic motivation. When parents are involved and resources are available, children receive structured guidance and support that reinforces their extrinsic motivation, helping them meet academic demands and lowering obstacles to staying engaged with tasks. These results align with previous studies that showed that parental guidance is a positive predictor of students’ intrinsic motivation to enhance their English proficiency ( Fan & Williams, 2010). Additionally, parents’ emotional and behavioral involvement has a deeper impact on children’s motivation and achievements ( Kim et al., 2020). Parents’ involvement in their children’s English learning is strongly associated with the children’s English proficiency ( Butler & Le, 2018). Support provided by parents at home plays a crucial role in boosting YLs’ academic performance ( Tan & Caleon, 2022; Torrecilla & Hernández-Castilla, 2020). Furthermore, research has demonstrated that teacher-parent communication improves students’ academic performance ( Tan & Caleon, 2022), English learning motivation and engagement ( Fan & Williams, 2010). Thus, our findings are aligned with the results of these previous studies.

Conversely, enrichment and extracurricular activities provide meaningful, autonomy-supportive experiences that can ignite curiosity and enjoyment, thereby fostering intrinsic motivation. These results are in line with a previous study that found that higher SES parents can provide more frequent English use outside the classroom, which increasingly fostered their children’s competence and self-determined motivation ( Butler, 2015b). Parental expectations connect the two motivational dimensions: when expectations are high yet supportive, they can strengthen extrinsic goals while also conveying trust in the child’s abilities, which is a central source of intrinsic motivation. Parental expectations were positively correlated to learners’ English proficiency ( Butler & Le, 2018), achievements ( Tan & Caleon, 2022), engagement levels, and intrinsic motivation in English ( Fan & Williams, 2010). Overall, the identified factors show how parental engagement functions across both motivational dimensions, influencing children’s willingness to learn, their confidence, and their ongoing interest in English learning.

The CFA results suggest the model fits well, as indicated by fit indices: CFI = .945, TLI = .934, SRMR = .045, RMSEA = .059 ( Hu & Bentler, 1999). Despite a lower figure of AVE of parental expectation, it suggests adequate convergent validity ( Fornell & Larcker, 1981). With Cronbach’s alpha and composite reliability generally over .70 ( Tavakol & Dennick, 2011), the questionnaire demonstrates reliability, especially in the parental expectations construct. This includes high correlations among the items, effectively capturing their respective factors.

Thus, the questionnaire is both valid and reliable for measuring parents’ roles in influencing children’s motivation and task engagement and provides a strong basis for its interpretation and use. Grounded in self-determination theory ( Ryan & Deci, 2020), it captures how parents shape intrinsic and extrinsic motivation. In primary school English learning, where parental involvement, resources, enrichment, and expectations may differ, the questionnaire measured how engagement patterns support or hinder children’s motivation and task engagement.

In summary, the results can guide practice. Schools can design family engagement programs that strengthen motivational support. Policymakers can develop guidelines to promote equitable access to learning resources and extracurricular activities. Integrating assessment accuracy with theoretical and practical relevance, this questionnaire can become a powerful instrument for research and targeted interventions in children’s motivation and engagement.

## Conclusions

The new questionnaire intended to evaluate what roles parents play in their children’s English language learning motivation and task engagement. The instrument we validated shows robust psychometric qualities in both exploratory and confirmatory factor analyses. The five-factor model comprising (1) parental involvement in their offsprings’ language learning, (2) parents’ expectations about their success, and three additional factors related to what parents make available to their children, including access (3) to resources, (4) enrichment activities, and (5) extracurricular activities was validated by satisfactory model fit indices, suggesting a well-fitting measurement model. Strong convergent validity and consistently high reliability measures also confirm that the constructs are assessed both accurately and reliably. These results indicate that the questionnaire can serve as a valid and reliable instrument for investigating the impact of different aspects of parental support on fifth graders’ language learning motivation and engagement with various tasks related to learning English.

Furthermore, the model is in line with what authors of theoretical models (e.g., Lamb, 2017; Mihaljević Djigunović & Nikolov, 2019; Ryan & Deci, 2020) practical advice for stakeholders (e.g., Butler, 2015a), and teachers’ handbooks recommended as well as with empirical research included in our literature review on the ways in which parents can support their children during their language learning journey.

Although the instrument demonstrated both validity and reliability within Indonesian primary schools, it has some limitations. First, its broader applicability may be constrained due to differing parental roles and expectations across various cultural, socio-economic, and educational backgrounds. Additionally, the use of self-reported data introduces the potential for social desirability bias. To enhance its cross-cultural relevance, future research should involve a range of viewpoints and pilot the instrument in new contexts with a wider range of age groups of YLs.

## Implications

This study has some implications. First, for practice and language policies, this research provides an effective diagnostic tool for teachers, school leaders, and policymakers to pinpoint and understand the particular dimensions of parental involvement that affect children’s motivation and engagement activities that are conducive to their learning of English. By identifying strengths and weaknesses in parental involvement, resources, or expectations, targeted interventions and family engagement initiatives can be designed to improve student success. Schools can create workshops or allocate resources that align with parents’ contributions to supporting their children’s English learning.

Pedagogically, the study highlights the critical roles of recognising parents as active collaborators in the educational journey. Educators can modify communication methods and classroom activities to better align with the most influential types of parental support, like promoting enrichment activities or engaging parents in extracurricular events. Additionally, awareness of the differing parental expectations can help educators improve student learning experiences, foster motivation and engagement.

Theoretically, the study improves our understanding of the impact of parental behaviour in educational psychology and motivation studies. By supporting a multidimensional view of parental roles, findings underscore that family support is diverse, consisting of separate yet interconnected aspects. The outcomes are consistent with self-determination theory, as it provides a more nuanced perspective to explore how different forms of parental participation may impact student motivation and task engagement in learning English.

## Ethical approval

We obtained ethical approval from The Doctoral School of Education’s Institutional Review Board at the University of Szeged (Reference number: 24/2023).

## Informed consent

All adult participants gave written informed and voluntary consent.

## Consent for publication

This manuscript is unpublished and not under review by another journal.

## Data Availability

The data cannot be shared publicly due to restrictions for ethical and security reasons. The IRB of the doctoral school of education at the University of Szeged did not allow us to share our data with a third party. The datasets from this study can be requested from the corresponding author at maisrilena@fip.unp.ac.id Figshare: *Parents’ roles in young learners’ motivation and task engagement in Indonesian primary schools*: *Questionnaire development and validation.* https://doi.org/10.6084/m9.figshare.30302089 ( Lena, 2025a). The project contains the following extended data:
- Parents’ questionnaire Data are available under the terms of the CC BY 4.0 Figshare: *Parents’ roles in young learners’ motivation and task engagement in Indonesian primary schools*: *Questionnaire development and validation.* https://doi.org/10.6084/m9.figshare.30302086 ( Lena, 2025b). The project contains the following extended data:
- Consent form for participation Data are available under the terms of the CC BY 4.0
