# Supplementary material for: Parents’ roles in young learners’ motivation and task engagement in Indonesian primary schools: Questionnaire development and validation
Source: F1000Res. 2025 Dec 24;14:1127. Originally published 2025 Oct 17. [Version 2] doi: 10.12688/f1000research.170391.2 (PMC12669978; doi:10.12688/f1000research.170391.2)
Supplement: Peer review history

# Reviewer report: Reviewer response for version 1

**DOI:** 10.5256/f1000research.187839.r427999

**Published:** 2025-12-01

**Authors:** Ümit Ünsal Kaya

**Affiliations:** 1 Afyon Kocatepe University, Afyonkarahisar, Turkey

**Evaluation of the Article**

**1. Scientific Soundness**

 The research is methodologically rigorous and theoretically grounded in self-determination theory. The factor analyses are appropriately applied and clearly reported. The reliability coefficients and fit indices demonstrate that the instrument is both valid and reliable for its intended purpose. The study aligns with best practices in educational measurement and questionnaire development.

**2. Originality and Contribution**

 This work provides an important contribution to the field of language education and parental involvement studies by developing the **first validated instrument** tailored to the Indonesian context. The focus on parents of primary school children learning English as a foreign language is both timely and valuable, particularly in light of recent curricular changes in Indonesia. The study has both theoretical implications—by extending motivation research into underrepresented contexts—and practical applications for educators and policymakers.

**3. Writing and Structure**

 The article is well-structured and clearly written. The academic tone is appropriate, and the progression of ideas is logical and coherent. The introduction thoroughly reviews relevant literature, the methodology section is detailed and transparent, and the discussion links findings back to theoretical and practical concerns. Tables and figures are informative and well-integrated into the text.

**4. Language Quality**

 The language is clear and fluent, with no notable grammatical or typographical issues. Terminology is used appropriately throughout, and the academic register is consistently maintained.

**Final Recommendation**

 This article is scientifically sound, well-written, and makes a valuable contribution to the field. I find **no weaknesses** that require correction or revision.

Is the work clearly and accurately presented and does it cite the current literature?

Yes

If applicable, is the statistical analysis and its interpretation appropriate?

Yes

Are all the source data underlying the results available to ensure full reproducibility?

Yes

Is the study design appropriate and is the work technically sound?

Yes

Are the conclusions drawn adequately supported by the results?

Yes

Are sufficient details of methods and analysis provided to allow replication by others?

Yes

Reviewer Expertise:

child development, early childhood education

I confirm that I have read this submission and believe that I have an appropriate level of expertise to confirm that it is of an acceptable scientific standard.

# Reviewer report: Reviewer response for version 1

**DOI:** 10.5256/f1000research.187839.r428006

**Published:** 2025-11-11

**Authors:** Pawel Larionow

**Affiliations:** 1 University College of Professional Education, Wrocław, Poland

Dear authors,

 I have reviewed your paper and I am providing my comments below: 

 Abstract

 1. I think this statement is too strong. "No research has been conducted in the Indonesian context on what roles parents play in their children’s motivation and task engagement.". In the background section, there is no aim of the study.

 2. I believe that this statement is unclear. "Research questions examined factors affecting its effectiveness.".

 3. Jamovi is a statistical program. It is a name, and it should be written with a capital letter.

 4. The Conclusions are too broad and specifying them seems to be a good choice.

 Introduction

 5. I think that starting the introduction with the sentence "The study aims to develop, pilot, and validate a questionnaire on the role of parents in motivating and engaging young learners with the tasks assigned by teachers in their English classes." is uncommon.

 6. I am not very familiar with the theory of this particular topic, and I would prefer to ask editors and other more experienced reviewers to analyze this section.

 7. I find this sentence unclear "1. What are the underlying factors that structure the questionnaire measuring parents’ roles in their children’s English learning motivation and task engagement?".

 Method

 8. Several sections are overlapping. For instance, Context of the study, Research design and Procedure can be combined. Please carefully reconsider the paper structure.

 9. Several statements are vague. For instance,  "We developed the parents’ role questionnaire because the existing questionnaire did not relate to the study’s purposes."

 10. English should be edited as there are several difficult to read statements.

 11. The development of a questionnaire and its all standards procedures were not described sufficiently. Please elaborate on these, and indicate details. For instance, how many parents did participate in a pilot testing? And other aspects.

 12. The description of statistical analyses was not provided in the Method section. The authors added several description in the Results section, but this should have been done in the Methods section.

 Results

 13. All factor loadings for all items were not mentioned. Please add these to assess potential cross-loadings.

 14. The sentences below may indicate that the scale does not have sufficient discriminative power as all items have values above 3 and even around the maximum value of 4. "Table 7 presents the descriptive statistics for the dimensions of parental roles, indicating generally high levels of agreement across all items, with means fluctuating between 3.06 and 3.73 on a 4-point scale. The highest mean scores were found in parental expectations (M = 3.65 to 3.73), reflecting strong convictions regarding the importance of English and significant aspirations for their children’s English proficiency.".

 15. The convergent and divergent validity seems not to have been assessed.

 Discussion

 16. The Discussion is very short, basically repeating the results from the Results section. The discussion should be elaborated. Please discuss theoretical and practical value of this study. Limitations of the study were not indicated.

Is the work clearly and accurately presented and does it cite the current literature?

Partly

If applicable, is the statistical analysis and its interpretation appropriate?

Partly

Are all the source data underlying the results available to ensure full reproducibility?

No

Is the study design appropriate and is the work technically sound?

Partly

Are the conclusions drawn adequately supported by the results?

Partly

Are sufficient details of methods and analysis provided to allow replication by others?

Partly

Reviewer Expertise:

psychological assessment, psychopathology, emotion, emotion regulation, psychometrics

I confirm that I have read this submission and believe that I have an appropriate level of expertise to confirm that it is of an acceptable scientific standard, however I have significant reservations, as outlined above.

# Reviewer report: Reviewer response for version 1

**DOI:** 10.5256/f1000research.187839.r425669

**Published:** 2025-11-11

**Authors:** Panaoura Rita

**Affiliations:** 1 Frederick University, Nicosia, Cyprus

Introduction

 1. The abstract is generally clear and well structured, effectively presenting the study’s rationale, methods, results, and implications. It highlights a relevant gap—the lack of validated tools assessing parents’ influence on children’s motivation and task engagement in Indonesia—and clearly describes the process of instrument development and validation.

 Introduction

 2. The introduction provides a solid and logically organized foundation for the study. It effectively establishes the educational and policy context of English language learning in Indonesian primary schools and clearly articulates the relevance of investigating parental influence on young learners’ motivation and task engagement. The integration of national regulations and curricular details strengthens the argument’s contextual grounding. In my opinion, the section could benefit from a smoother narrative flow. Some parts read as a list of facts rather than a cohesive argument leading to the research gap.

 3. The final paragraphs clearly define the study’s novelty and research questions, providing a convincing rationale for the questionnaire’s development.

 Literature review

 4. The literature review is comprehensive and demonstrates strong engagement with recent and relevant sources. The discussion of socioeconomic and educational factors provides useful empirical grounding, while the integration of self-determination theory and engagement constructs shows theoretical awareness. The section tends to summarize studies descriptively rather than critically, offering limited synthesis or comparison among findings. It could better highlight inconsistencies or contextual differences between Western and Asian research traditions, reinforcing the need for a culturally adapted Indonesian instrument.

 5. The explanation of existing questionnaires is informative but somewhat detailed; summarizing overlapping methodological information would improve conciseness.

 Method

 6. The Method section is well-structured and provides sufficient procedural detail for replication. The section could be strengthened by briefly justifying the use of convenience sampling and discussing its potential implications for generalizability. Additionally, the authors might clarify how expert feedback specifically informed item revision and whether any items were removed or reworded after piloting.

 7. A table can be presented at the end of a paragraph. Do not split the paragraph or the sentence by introducing a table.

 Results

 8. An introduction paragraph is needed to explain the presentation of the results in respect to the posed research questions.

 9. The section reads somewhat mechanically, with limited interpretation of what the numerical outcomes imply in practical or conceptual terms. For instance, brief comments linking the five extracted factors to parental behaviours or engagement mechanisms would help readers connect results to real-world meaning.

 10.Some redundancy could be reduced by merging overlapping explanations of fit indices in EFA and CFA.

 Discussion

 11. I have been disappointed by the specific section. It is not a section, it is only a paragraph.  The Discussion section is notably underdeveloped, as it offers only a brief restatement of statistical results without providing genuine interpretation or theoretical insight. Rather than simply confirming that the instrument is valid and reliable, this section should have explored what the findings reveal about the multifaceted roles of Indonesian parents in supporting their children’s English learning.

 12. The study’s alignment with self-determination theory could have been elaborated to explain how different parental behaviours foster intrinsic versus extrinsic motivation.

 13. Expanding the discussion to include theoretical interpretation, contextual relevance, and implications for educators and policymakers would transform it from a descriptive summary into a meaningful scholarly dialogue.

 Conclusions

 14. The Conclusions section provides a clear summary of the study’s outcomes, confirming the questionnaire’s validity and reliability and restating its five-factor structure. However, it primarily reiterates statistical findings rather than synthesizing their broader significance. A stronger conclusion would integrate theoretical, practical, and contextual implications, emphasizing how the validated tool advances understanding of parental roles in children’s motivation and task engagement, particularly within Indonesia’s evolving English education landscape.

 15. The authors could also highlight how the instrument may inform teacher training, parental engagement programs, or policy decisions aimed at fostering home–school collaboration

Is the work clearly and accurately presented and does it cite the current literature?

Partly

If applicable, is the statistical analysis and its interpretation appropriate?

Yes

Are all the source data underlying the results available to ensure full reproducibility?

Yes

Is the study design appropriate and is the work technically sound?

Yes

Are the conclusions drawn adequately supported by the results?

No

Are sufficient details of methods and analysis provided to allow replication by others?

Yes

Reviewer Expertise:

Mathematics Education, self-regulation, parental involvement, inquiry based methods, digital tools in education

I confirm that I have read this submission and believe that I have an appropriate level of expertise to confirm that it is of an acceptable scientific standard, however I have significant reservations, as outlined above.
